# TNF-α acutely enhances acid-sensing ion channel currents in rat dorsal root ganglion neurons via a p38 MAPK pathway

**DOI:** 10.1186/s12974-021-02151-w

**Published:** 2021-04-14

**Authors:** Shuang Wei, Chun-Yu Qiu, Ying Jin, Ting-Ting Liu, Wang-Ping Hu

**Affiliations:** 1 Research Center of Basic Medical Sciences, School of Basic Medical Sciences, Hubei University of Science and Technology, 88 Xianning Road, Xianning, 437100 Hubei PR China; 2 Department of Pharmacology, Hubei University of Science and Technology, 88 Xianning Road, Xianning, 437100 Hubei PR China

**Keywords:** Tumor necrosis factor-α, Acid-sensing ion channels, Electrophysiology, Nociceptive response, Dorsal root ganglion neuron

## Abstract

**Background:**

Tumor necrosis factor-α (TNF-α) is a pro-inflammatory cytokine involved in pain processing and hypersensitivity. It regulates not only the expression of a variety of inflammatory mediators but also the functional activity of some ion channels. Acid-sensing ion channels (ASICs), as key sensors for extracellular protons, are expressed in nociceptive sensory neurons and contribute to pain signaling caused by tissue acidosis. It is still unclear whether TNF-α has an effect on functional activity of ASICs. Herein, we reported that a brief exposure of TNF-α acutely sensitized ASICs in rat dorsal root ganglion (DRG) neurons.

**Methods:**

Electrophysiological experiments on rat DRG neurons were performed in vitro and acetic acid induced nociceptive behavior quantified in vitro.

**Results:**

A brief (5min) application of TNF-α rapidly enhanced ASIC-mediated currents in rat DRG neurons. TNF-α (0.1-10 ng/ml) dose-dependently increased the proton-evoked ASIC currents with an EC_50_ value of 0.12 ± 0.01 nM. TNF-α shifted the concentration-response curve of proton upwards with a maximal current response increase of 42.34 ± 7.89%. In current-clamp recording, an acute application of TNF-α also significantly increased acid-evoked firing in rat DRG neurons. The rapid enhancement of ASIC-mediated electrophysiological activity by TNF-α was prevented by p38 mitogen-activated protein kinase (MAPK) inhibitor SB202190, but not by non-selective cyclooxygenase inhibitor indomethacin, suggesting that p38 MAPK is necessary for this enhancement. Behaviorally, TNF-α exacerbated acid-induced nociceptive behaviors in rats via activation of local p38 MAPK pathway.

**Conclusions:**

These results suggest that TNF-α rapidly enhanced ASIC-mediated functional activity via a p38 MAPK pathway, which revealed a novel peripheral mechanism underlying TNF-α involvement in rapid hyperalgesia by sensitizing ASICs in primary sensory neurons.

## Introduction

Tumor necrosis factor-α (TNF-α), a pro-inflammatory cytokine, plays a critical role in the development and maintenance of pain [1]. TNF-α is released by a variety of cells including inflammatory, immune, glia, and neuronal cells [2, 3]. And endogenous TNF-α levels increase under inflammatory and neuropathic pain conditions [4, 5]. Neutralizing endogenous TNF-α with antibodies attenuates thermal and mechanical hyperalgesia in neuropathic pain [6, 7]. Animals display higher sensitivity to thermal and mechanical stimuli after TNF-α is injected into the paw [4, 8, 9]. TNF-α regulates pain signaling through genomic and non-genomic mechanisms. On the one hand, TNF-α has long-lasting effects by regulating the expression of a variety of inflammatory mediators and modifying signaling proteins. On the other hand, TNF-α has also rapid onset effects by modulating the functional activity of a variety of ion channels. For example, TNF-α increases the expression of Na_v_1.7 channels, which are expressed in the cell bodies of neurons that act as nociceptive fibers Aδ and C [10, 11]. TNF-α has been shown to acutely increase tetrodotoxin (TTX)-resistant sodium channel currents in dorsal root ganglion (DRG) neurons [12, 13]. TNF-α also directly enhances the sensitivity of rat trigeminal neurons to capsaicin through a rapid non-genomic mechanism [14]. In addition, TNF-α regulates outward potassium channel currents, calcium channel currents, and hyperpolarization-activated cation currents in other neurons [15–18]. Studies have shown that peripheral administration of TNF-α rapidly enhances ongoing activity in nociceptive fibers, resulting in acute mechanical and thermal hypersensitivity [12, 19, 20]. Acute modulation of these ion channels by TNF-α can account for the rapid onset of TNF-α-induced pain hypersensitivity [21].

Besides the above ion channels, acid-sensing ion channels (ASICs) are also expressed in both DRG cell bodies and sensory terminals, where they assemble as homomeric or heteromeric channels containing three ASIC subunits to sense changes in extracellular pH [22–24]. Among seven ASIC subunits, ASIC3 subunit is the most abundant in DRG and has emerged as a critical pH sensor [25]. Proton is a canonical ligand for ASICs. It is released and causes tissue acidosis under multiple pathological conditions such as inflammation, tissue injury, ischemic stroke, and cancer [26–28]. Low pH (up to pH 6.0)-induced pain is significantly alleviated by non-selective ASIC inhibitor amiloride, suggesting that the pain sensation is mainly mediated by ASICs, but not by transient receptor potential vanilloid type 1 (TRPV1) [25, 29]. ASICs, especially ASIC3, are the major player in pain associated with tissue acidosis [25, 30–33].

In the present study, we investigated whether TNF-α had also a rapid effect on functional activity of ASICs in primary sensory neurons. We observed that a brief (5min) application of TNF-α rapidly enhanced ASIC-mediated currents in rat DRG neurons via a p38 mitogen-activated protein kinase (MAPK)-dependent pathway. The present studies provided a novel explanation for the rapid sensitization of pain induced by TNF-α.

## Materials and methods

### Isolation of DRG neurons

All experimental protocols were approved by the animal research ethics committee of Hubei University of Science and Technology (No. 2020-07). All procedures were made to minimize the sufferings of animals. Sprague-Dawley male rats (6- to 7-week-old) were anesthetized with 7% chloral hydrate. The DRGs were taken out and minced with fine spring scissors. The ganglion fragments were placed in a flask containing 5ml of Dulbecco’s modified Eagle’s medium (DMEM, Sigma). DMEM contained trypsin (type II-S, Sigma) 0.5 mg/ml, collagenase (type I-A, Sigma) 1.0 mg/ml, and DNase (type IV, Sigma) 0.1 mg/ml and was incubated at 35°C in a shaking water bath for 25–30 min. Soybean trypsin inhibitor (type II-S, Sigma) 1.25 mg/ml was then added to stop trypsin digestion. Freshly dissociated neurons were placed into a 35-mm Petri dish and kept for at least 1 h in normal external solution before the start of electrophysiological experiments.

### Electrophysiological recordings

Whole-cell patch clamp was carried out at room temperature (22–25°C) using a MultiClamp-700B amplifier and Digidata-1440A A/D converter (Axon Instruments, CA, USA). Dissociated neurons were placed into a 35-mm Petri dish and were bathed in an external solution containing (mM): NaCl 140, KCl 5, CaCl_2_ 2.5, MgCl_2_ 2, HEPES 10, MES 10, d-glucose 10. Its pH was adjusted to7.4 with NaOH and its osmolarity to 330 mOsm/L with sucrose. Cells were kept for at least 60 min in normal external solution before the start of electrophysiological experiments. The neurons selected for electrophysiological experiment were 15–35μm in diameter, which are thought to be nociceptive neurons [34]. Recording pipettes were pulled using a Sutter P-97 puller (Sutter Instruments, CA, USA). The micropipettes were filled with internal solution containing (mM): KCl 140, MgCl_2_ 2, HEPES 10, EGTA 11, ATP 4, and Na_2_GTP 0.3. Its pH was adjusted to 7.2 with KOH and its osmolarity to 310 mOsm/L with sucrose. The resistance of the recording pipette was in the range of 3–6MΩ. To establish a whole-cell configuration, a small patch of membrane underneath the tip of the pipette was aspirated to form a giga seal and then a negative pressure was applied to rupture it. The series resistance was compensated for by 70–80%. The adjustment of capacitance compensation was also done before recording the membrane currents. The membrane voltage was maintained at −60 mV in all voltage-clamp experiments. Current-clamp recordings were obtained by switching to current-clamp mode after a stable whole-cell configuration was formed in voltage-clamp mode. Only cells with a stable resting membrane potential (more negative than −50 mV) were used in the study.

### Drug application

Drugs were obtained from Sigma Chemical Co. (St. Louis, MO, USA) and used in the experiments which include hydrochloric acid, TNF-α, SB202190, indomethacin, amiloride, APETx2, capsaicin, and AMG 9810. Different pH values were configured with hydrochloric acid and external solution, and MES was used to buffer solution pH. Working TNF-α and other drugs were freshly prepared in normal external solution and held in a series of independent reservoirs. The pipette tips connecting reservoirs were positioned ∼30 μm away from the recorded neurons. The application of each drug was driven by gravity and controlled by the corresponding valve. To functionally characterize ASIC activity, we used AMG9810 (5 μM) to block TRPV1 in the extracellular solution [35].

### Nociceptive behavior induced by acetic acid in rats

Rats were placed in a 30 × 30 × 30 cm Plexiglas chamber and allowed to habituate for at least 30 min before nociceptive behavior experiments. A double-blind experiment was carried out. Separate groups of rats were coded and pretreated with 50 μl AMG 9810 (10 μM) together with vehicle, different doses of TNF-α, and TNF-α + SB202190 in ipsilateral hindpaw before the injection of acetic acid. After 5 min, the other observers, who were unaware of treatment allocation, subcutaneously administered acetic acid solution (1%, 50 μl) into the hind paw using a 30-gauge needle connected to a 100-μL Hamilton syringe. And nociceptive behavior (that is, number of flinches) was monitored for the next 5 min [25, 36]. To demonstrate that TNF-α produced enhancement of acetic acid-induced behaviors through a local effect in the hindpaw, another group of rats received an injection of acetic acid in one hindpaw and TNF-α (10 ng in 50μl) into contralateral hindpaw.

### Data analysis

We determined the normality of the data distribution and then used one-way analysis of variance (ANOVA), followed by Bonferroni’s post hoc test for normally distribution data. Data are expressed as mean ± S.E.M. *P* < 0.05 were considered statistically significant. Statistical tests were carried out with Graphpad Prism 4. Statistical analysis of concentration–response data was performed using nonlinear curve-fitting program ALLFIT. The data for proton is a good fit to the logistic equation *I* = *I*_max_/[1 + (pH_0.5_/pH)^*n*^], where pH is the pH value used, *I* is the normalized current response value, pH_0.5_ is the pH value for half-maximal current response, and *n* is the Hill coefficient.

## Results

### TNF-α acutely enhanced ASIC-mediated currents in rat DRG neurons

**Fig. 1:**
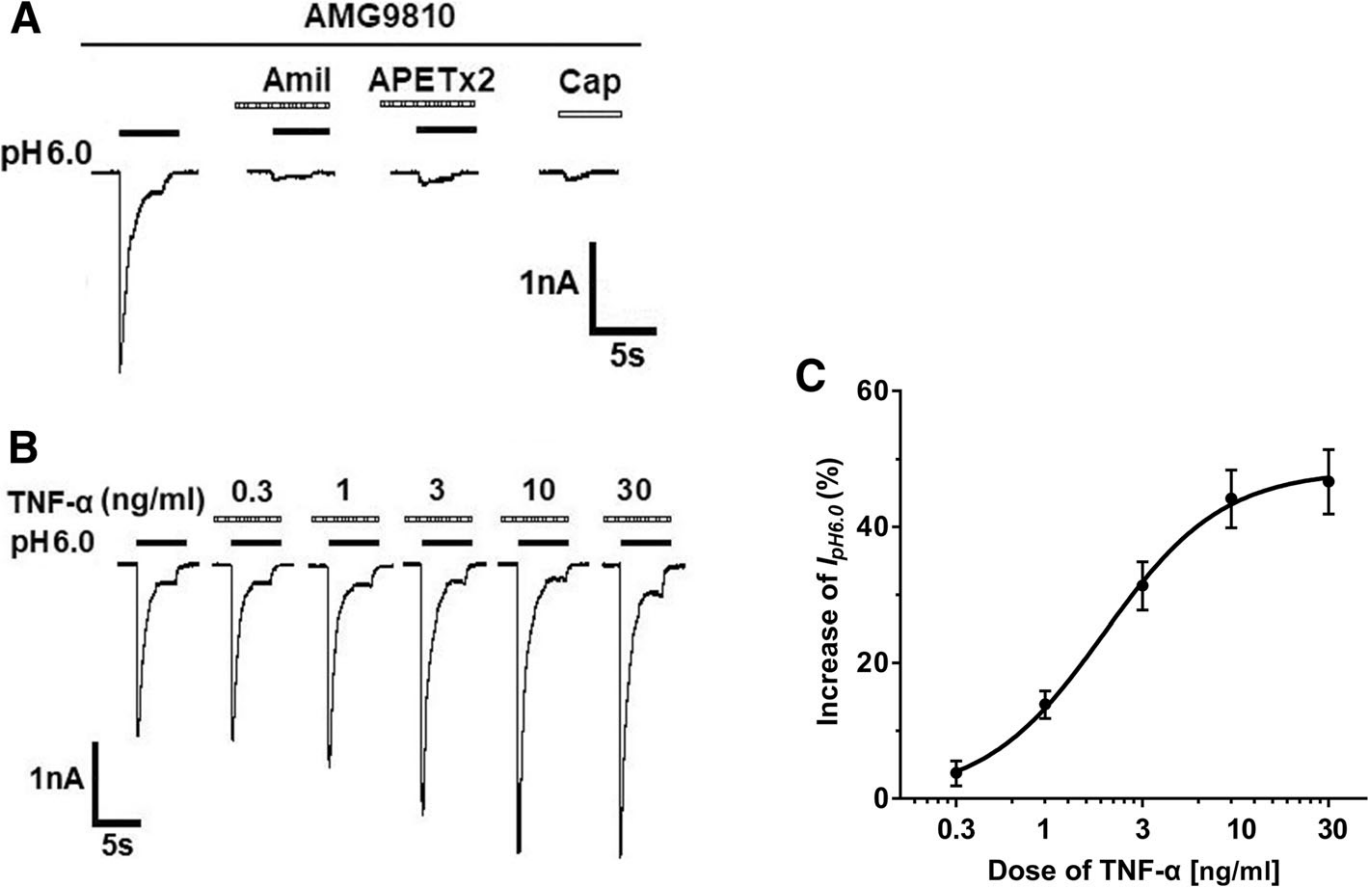
TNF-α acutely enhanced ASIC-mediated currents in rat DRG neurons. **a** Representative current traces were evoked by application of a pH 6.0 acidic solution for 5 s in a tested DRG neuron in the presence of AMG9810 (5 μM). The low pH-induced current (I_pH6.0_) could be blocked by broad-spectrum ASIC channel blocker amiloride (Amil, 10 μM) and ASIC3 blocker APETx2 (2 μM). Capsaicin (Cap, 100 nM) failed to evoke any membrane currents in the presence of AMG9810 (5 μM). All membrane potentials were clamped at −60 mV. B. The sequential current traces illustrated that the amplitude of *I*_pH6.0_ progressively increased after different doses of TNF-α was pre-treated to a representative DRG cell. C. The graph showed TNF-α dose-dependently increased *I*_pH6.0_ with an EC_50_ of 1.96 ± 0.15 ng/ml. Each point represents the mean ± SEM of 7–10 cells

In the present study, AMG9810 (5 μM) was added to external solution to block proton-induced TRPV1 activation. As shown in Fig. 1a, a sudden drop in extracellular pH from 7.4 to 6.0 produced a rapid inward current (*I*_*p*H6.0_) in DRG neurons. The *I*_pH6.0_ could be almost completely blocked by 10 μM of amiloride, a broad-spectrum ASIC channel blocker, and also by 2 μM APETx2, an ASIC3 blocker. In contrast, capsaicin (100 nM) failed to evoke any membrane currents in the presence of AMG9810. Thus, these acid-induced currents were considered to be ASIC currents or ASIC3-mediated currents after TRPV1 activation was blocked by AMG9810.

In some DRG neurons sensitive to acid stimuli, we first evaluated the effects of brief application of TNF-α on the ASIC currents. TNF-α was pre-incubated to DRG neurons for 5min prior to application of pH 6.0 acidic solution. As shown in Fig. 1b and c, a brief (5min) application of TNF-α acutely increased the peak amplitude of *I*_pH6.0_. The enhancement of *I*_pH6.0_ occurred 5min after the onset of TNF-α application. This enhancement of *I*_pH6.0_ was dependent upon the doses of TNF-α treatment. In a representative DRG neuron, the peak amplitude of *I*_pH6.0_ progressively increased as doses of pre-treated TNF-α increased from 0.3 to 30 ng/ml (Fig. 1b). Figure 1c shows the dose-response curve for TNF-α with an EC_50_ (half-maximal effective dose) value of 0.12± 0.01 nM (1.96 ± 0.15 ng/ml). The results indicated that TNF-α rapidly enhanced ASIC currents in rat DRG neurons in dose-dependent manner.

**Fig. 2:**
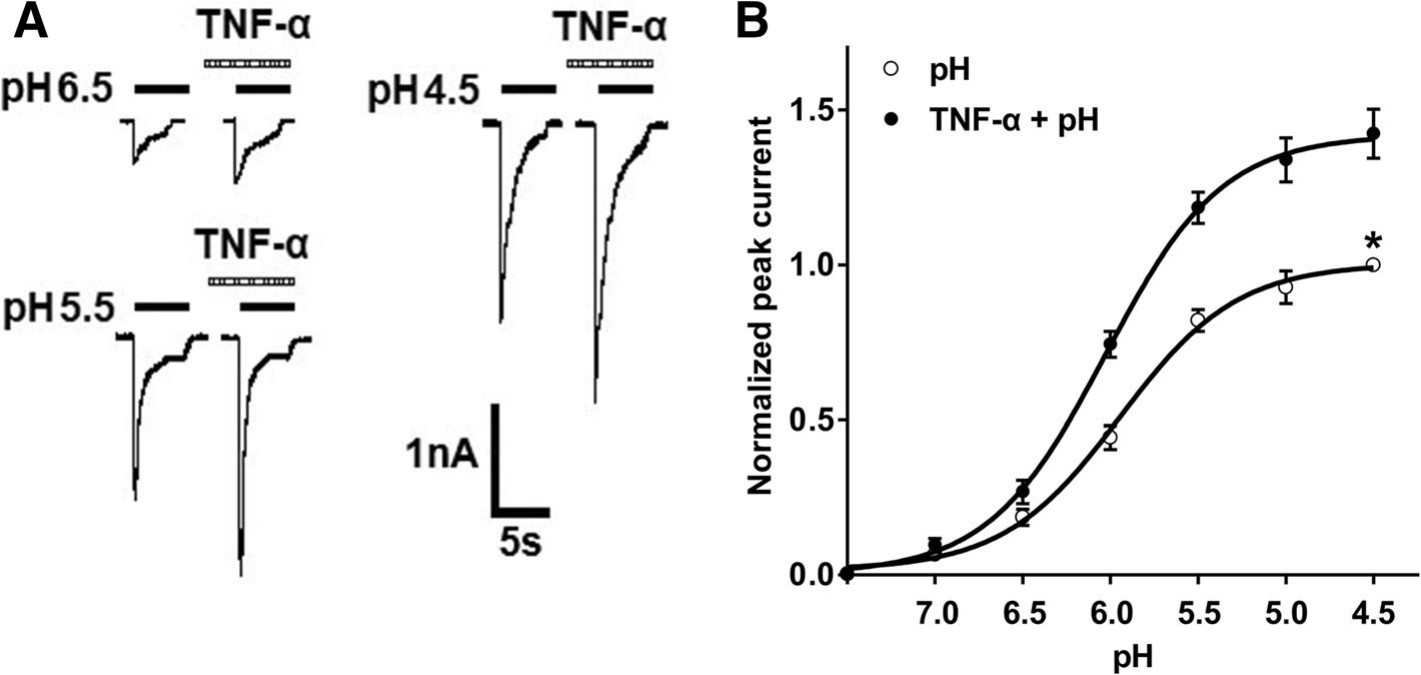
TNF-α shifted upwards the concentration-response curve for protons. **a** Sequential currents were evoked by three different low pH values in the absence and presence of 10 ng/ml TNF-α pretreatment. **b** Concentration-response curves for protons in the absence (○, white circle) and presence (●, black circle) of TNF-α. Concentration-response curve for protons shifted upwards in the presence of extracellular TNF-α (10 ng/ml). Each point represents the mean ± S.E.M. of 7–10 DRG neurons from 4–6 rats. All peak current values were normalized to the peak current maximally activated by pH 4.5 applied alone in the absence of TNF-α (marked with asterisk). The figure shows averaged data fitted with the Hill equation. The curves shown are a best fit of the data to the logistic equation *I* = *I*_max_/[1 + (pH_0.5_/pH)^*n*^], where pH is the pH value used, *I* is the normalized current response value, pH_0.5_ is the pH value for half-maximal current response, and *n* is the Hill coefficient. The Hill coefficients for the cases with and without TNF-α pre-application were 1.32 and 1.30, respectively

We then investigated the effects of TNF-α on concentration-response curve for protons. ASIC currents were measured by applying a range of different pH values in the absence and presence of TNF-α. Figure 2a shows that peak amplitudes of *I*_pH6.5_, *I*_pH5.5_, and *I*_pH4.5_ increased after pre-application of 10 ng/ml TNF-α for 5 min. Figure 2b shows concentration-response curve for protons shifted upwards by TNF-α treatment. First, TNF-α caused an increase of 42.34 ± 7.89% in the maximal current response to pH 4.5. Second, the Hill coefficient or slope of two curves had not significant difference in the absence and presence of TNF-α (pH: *n* = 1.30 ± 0.19; TNF-α + pH: *n* = 1.32 ± 0.21; *P* > 0.1, post hoc Bonferroni’s test). Third, the pH_0.5_ (pH for half-maximal activation) values of two curves had also no statistical difference (pH: pH_0.5_ = 5.94 ± 0.12; TNF-α + pH: pH_0.5_ = 6.03 ± 0.16; *P* > 0.1, *post hoc* Bonferroni’s test). We therefore concluded that sensitization of ASICs by TNF-α was not due to a change in the apparent affinity of ASICs for protons.

### TNF-α-induced enhancement of ASIC currents was mediated by p38 MAPK, but not cyclooxygenase

**Fig. 3:**
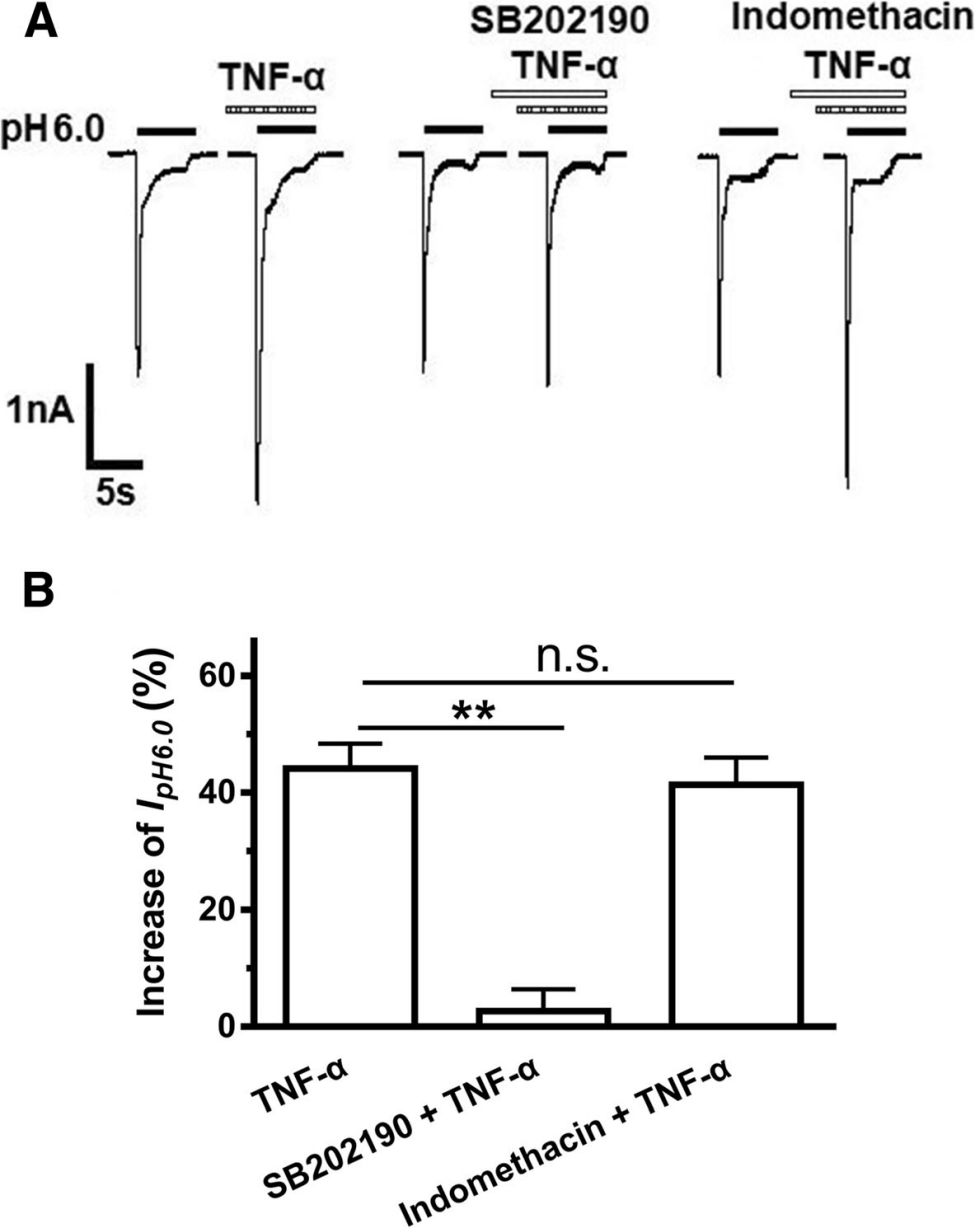
p38 MAPK, but not COX, was required for TNF-α-induced enhancement of ASIC currents. Representative current traces in (**a**) and the bar graph in (**b**) showed that the effects of TNF-α (10 ng/ml) alone, p38 MAPK inhibitor SB202190 plus TNF-α, and non-selective COX inhibitor indomethacin plus TNF-α on pH 6.0 acid- induced currents. *I*_*pH6*.0_ was enhanced by TNF-α (10 ng/ml) pre-applied alone for 5 min, and the TNF-α (10 ng/ml) enhancement of *I*_pH6.0_ was blocked by the pre-treatment of SB202190 (10 μM, 3+5=8 min), but not by the pre-treatment of indomethacin (30 μM, 3+5=8 min). Statistical tests were performed using one-way ANOVA followed by post hoc Bonferroni’s test, and significance is shown **P < 0.01. n.s. Not significant. *n* = 6 in each column

We further explored the pathway linking TNF-α to its effect on ASIC currents. It has been demonstrated that TNF-α can signal through activation of p38 MAPK in DRG neurons as well as many other cell types [12, 37, 38]. In addition, TNF-α can induce functional expression of cyclooxygenase (COX)-2 in cultured DRG neurons [39]. We therefore investigated the roles of p38 MAPK and COX in the enhancement of ASIC currents by TNF-α. As shown in Fig. 3a and b, the amplitude of *I*_pH6.0_ increased 44.14 ± 4.26% by TNF-α (10 ng/ml) pre-treatment alone. SB202190, a fast-acting p38 MAPK inhibitor, was applied to DRG neurons for 3 min followed by mixture of SB202190 and TNF-α for additional 5 min. The pretreatment of SB202190 (10 μM) substantially prevented the TNF-α-mediated increase in ASIC currents, and the amplitude of *I*_pH6.0_ increased only 2.67 ± 3.71% (*P* < 0.01, compared with TNF-α pretreatment alone, one-way ANOVA followed by post hoc Bonferroni’s test, *n* = 6; Fig. 3a and b). Indomethacin, a potent inhibitor for both COX-1 and COX-2, was also applied to DRG neurons similar to SB202190 treatment. In contrast, indomethacin failed to change TNF-α-mediated increase in ASIC currents (Fig. 3a and b). In addition, SB202190 or indomethacin alone had no effect on *I*_pH6.0_ (data not shown). These results indicated that p38 MAPK, but not COX, is necessary for TNF-α-induced enhancement of ASIC currents.

### TNF-α increased acid-evoked action potentials in rat DRG neurons

**Fig. 4:**
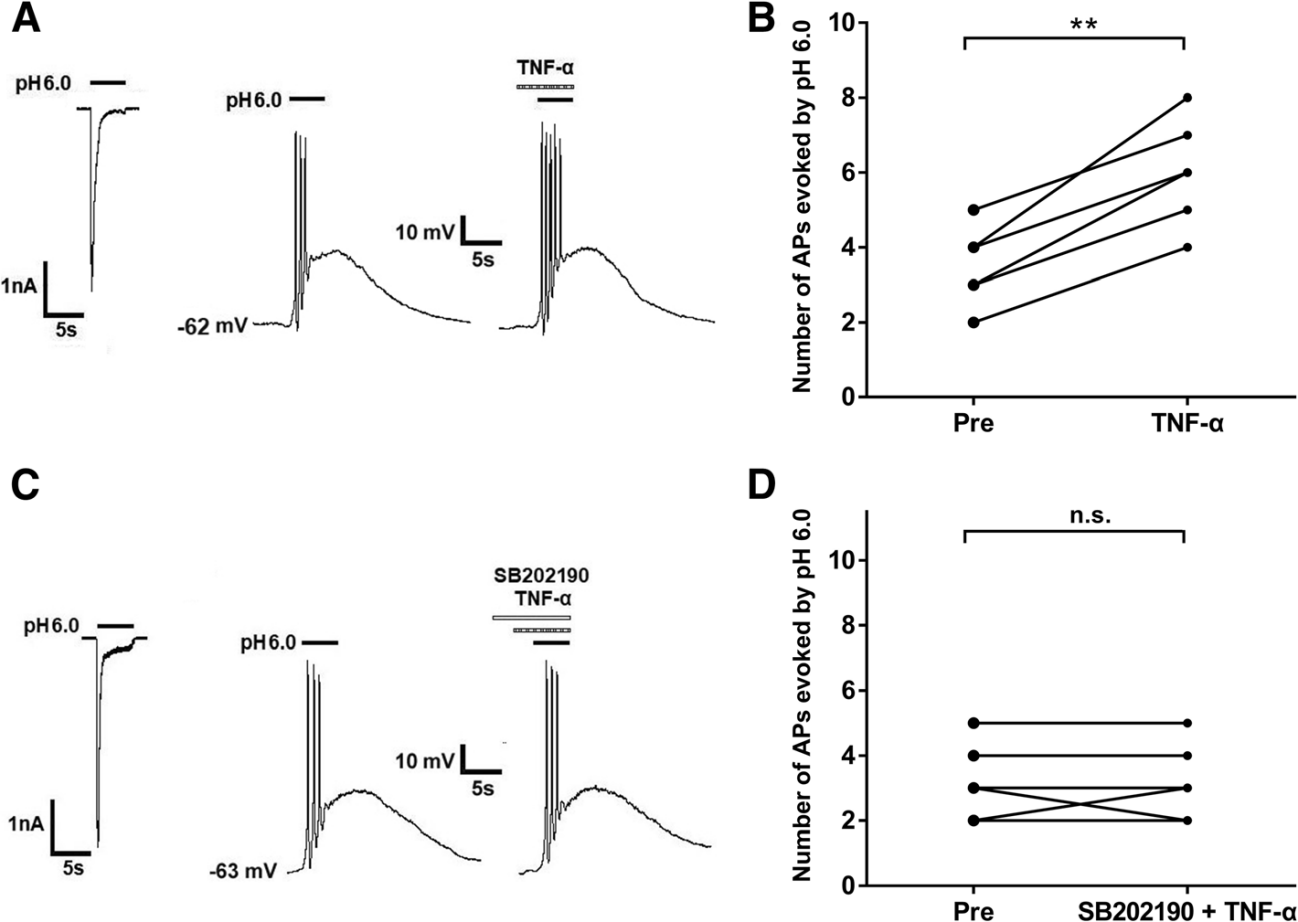
TNF-α increased acid-evoked action potentials in rat DRG neurons. **a**, **c** In the same DRG neuron, a pH 6.0 acidic stimulus induced not only a rapid inward current with voltage-clamp recording, but also action potential burst under current-clamp condition. Original action potentials (APs) were recorded before and after application of TNF-α (10 ng/ml, 5 min) alone (A) or co-application of both TNF-α (10 ng/ml, 5 min) and SB202190 (10 μM, 3+5=8 min) (C). AMG9810 (5 μM) was used to block proton-induced TRPV1 activation. B and D. The graphs showed the number of acid-evoked APs increased by pre-application of TNF-α alone, but not by co-application of both TNF-α and SB202190. **P < 0.01, paired *t*-test, *n* = 6 cells

ASICs are non-selective cation channels, once activation by acidification, which leads to membrane potential depolarization and neuronal excitation. We further observed whether TNF-α had effects on acid-evoked action potentials of rat DRG neurons. Although proton-induced TRPV1 activation was blocked in the presence of 5 μM AMG9810, we observed that an acid stimulus of pH 6.0 induced not only a rapid inward current with voltage-clamp recording, but also bursts of action potentials (APs) under current-clamp condition in the same DRG neuron (Fig. 4a and c). Consistent with that observed under voltage-clamp conditions, TNF-α pre-treatment also acutely increased the number of APs evoked by acidic stimuli of pH 6.0 in DRG neurons (Fig. 4a and b). In six DRG neurons treated with TNF-α (10 ng/ml for 5 min), the number of APs evoked by acidic stimuli of pH 6.0 significantly increased (*P* < 0.01, paired *t*-test, *n* = 6, Fig. 4b). In other six DRG neurons pre-treated with SB202190 (10 μM), TNF-α failed to increase the number of APs (*P* > 0. 1, paired *t*-test; *n* = 6, Fig. 4c and d). These results indicated that TNF-α also rapidly enhanced acid-evoked APs via a p38 MAPK-dependent pathway.

### TNF-α exacerbated acid-induced nociceptive behaviors in rats

**Fig. 5:**
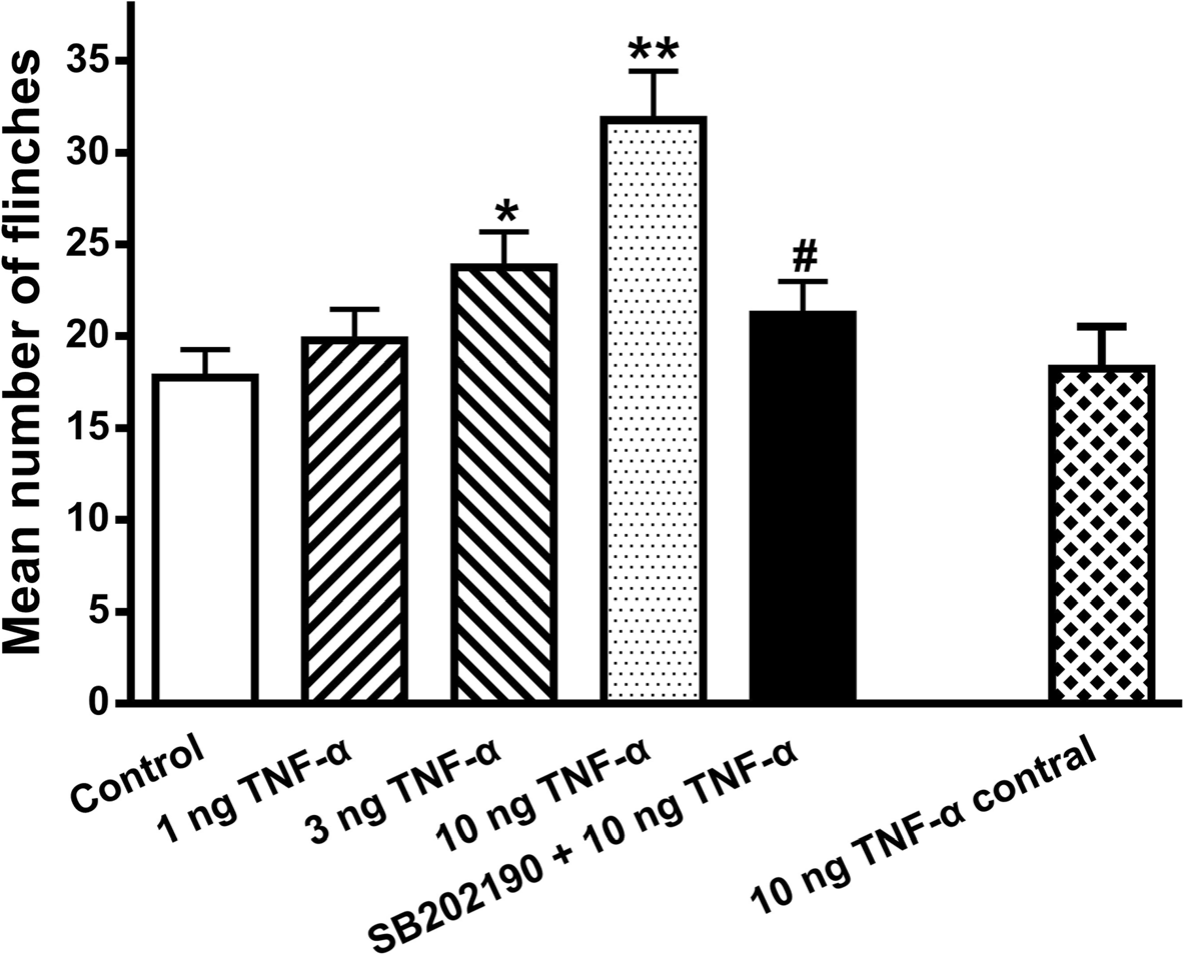
TNF-α exacerbated acid-induced nociceptive behaviors in rats. Nociceptive responses were evoked by intraplantar injection of acetic acid (1%, 50 μl) in rats in the presence of the TRPV1 inhibitor AMG9810 (10 μM). Intraplantar pretreatment of TNF-α (1, 3, and 10 ng) dose-dependently increased the number of acid-induced flinching. TNF-α (10 ng) exacerbating effect on nociceptive behaviors was reversed by intraplantar co-treatment of p38 MAPK inhibitor SB202190 (25 μM). Rats (10 ng TNF-α contral group) injected with acetic acid into one hind paw and TNF-α (10 ng) into the contralateral hind paw displayed similar nociceptive behaviors to those seen in control rats. Each bar represents the number of flinches that animals spent licking/lifting the injected paw during first 5-min observation period. *P < 0.05, **P < 0.01, one-way ANOVA followed by post hoc Bonferroni’s test, compared with control column; # *P* < 0.05, one-way ANOVA followed by post hoc *Bonferroni’s* test, compared with 10 ng TNF-α column. Each group represents the mean ± S.E.M. of 10 rats

Our above electrophysiological studies showed that TNF-α acutely sensitized ASICs via activation of p38 MAPK in vitro. We further ascertained whether TNF-α had effects on ASIC-mediated nociceptive behaviors through interacting with ASICs in vivo. Our previous studies observe that intraplantar injection of acetic acid into rats elicits ASIC-mediated nociceptive behaviors even if AMG 9810 (10 μM) blocked the activation of TRPV1 [40, 41]. We found that pretreatment with TNF-α (1, 3, and 10 ng in 50μl) dose-dependently exacerbated the acid-induced nociceptive behaviors (*p* < 0.05 and 0.01, one-way ANOVA followed by post hoc Bonferroni’s test, *n* = 10; Fig. 5). However, the aggravating effect of TNF-α on acid-induced nociceptive behaviors was prevented in rats, which were co-treated with the p38 MAPK inhibitor SB202190 (25μM in 50μl). The mean number of flinches in these rats significantly decreased, compared with that observed in rats pretreated with TNF-α (10 ng in 50μl) alone (*p* < 0.01, one-way ANOVA followed by post hoc Bonferroni’s test, *n* = 10; Fig. 5). In addition, injection of TNF-α (10 ng in 50μl) into the contralateral paws did not change acid-induced nociceptive behaviors. These results indicated that TNF-α exacerbated acid-induced nociceptive behaviors in rats via activation of local p38 MAPK pathway.

## Discussion

This study examined the effects of a brief exposure of TNF-α on functional activity of ASICs. We demonstrated that acute application of TNF-α rapidly enhanced ASIC-mediated and acid-evoked currents and action potentials in dissociated rat DRG neurons through a p38 MAPK-dependent mechanism. Behaviorally, TNF-α also exacerbated acid-induced nociceptive responses in rats via activation of local p38 MAPK pathway.

After proton-induced TRPV1 activation was block by AMG9810, capsaicin failed to induce any membrane currents. Thus, the low pH-evoked currents may be mediated by only ASICs in the present study. The conclusion was confirmed by the results that these currents were completely blocked by ASIC channel blocker amiloride and ASIC3 blocker APETx2. It has been found that there are seven ASIC subunits in DRG neurons, of which ASIC3 subunit is the most abundant [25]. We therefore considered that these low pH-evoked currents may be ASIC or ASIC3-like currents, although precise ASIC subunits need to be identified.

The present study showed that TNF-α can rapidly sensitized ASICs. A brief (5min) exposure of TNF-α dose-dependently enhanced ASIC currents in rat DRG neurons. TNF-α shifted upwards concentration-response curve for protons with a significant increase in the maximal current response to protons, whereas apparent affinity of ASICs for proton did not change. The rapid pH drop caused an activation of ASICs which promptly depolarized the membrane and initiated a burst of APs. The burst ended quickly because of prolonged depolarization which inactivated the sodium current [42]. The present study showed that a brief exposure of TNF-α also rapidly increased the number of action potentials evoked by low pH in current-clamp experiments. Obviously, the two results in current-clamp and voltage-clamp recordings corroborated each other. Together, TNF-α can rapidly exert an enhancing effect on the electrophysiological activity of ASICs in rat DRG neurons. This is consistent with earlier results showing that an application of TNF-α has an acute influence on ion channel currents including TTX-resistant Na^+^ currents, L-type Ca^2+^ currents, and TRPV1 currents [12, 14, 21].

TNF-α exerts its biological effects by associating with two tumor necrosis factor receptors: TNFR-1 and TNFR-2, which are expressed in DRG neurons [43–46]. TNFR-1 is shown to be involved in the enhancement of TTX-resistant Na^+^ currents [12]. TNFR1 activates multiple signaling pathways including ceramide signaling and activation of several MAPK pathways [37, 38]. It has been shown that TNF-α acutely regulates TTX-resistant Na^+^ channel currents and hyperpolarization-activated cation currents via a p38 MAPK pathway [12, 17, 18]. The present data showed that p38 MAPK may underlie the acute sensitization of ASICs by TNF-α, since administration of a p38 MAPK inhibitor SB202190 prevented the TNF-α-induced enhancement not only in ASIC-mediated electrophysiological activity *in vivo* but also in acid-induced nociceptive behaviors in vitro. While acute exposure (5min) of TNF-α enhanced ASIC currents, the effects were likely to result from a posttranslational modification of ASICs by TNF-α, such as phosphorylation. It has been shown that activation of p38 MAPK increases TTX-resistant sodium channel currents by phosphorylation of L1 loop serines of Nav1.8 channels [47]. Future work should examine whether ASICs can be phosphorylated by p38 MAPK.

It has been shown that TNF-α leads to COX-2-dependent production of prostaglandin E2 [48, 49]. Prostaglandin E2 increases Nav1.8 current density in a protein kinase A (PKA)- and protein kinase C (PKC)-dependent manner [50, 51]. Application (for 15 min) of TNF-α suppresses sustained potassium current in rat sensory neurons, via stimulating the synthesis and release of endogenous prostaglandins [52]. Our recent study also shows that prostaglandin E2 potentiates ASIC currents via intracellular PKC and PKA signaling pathways [40]. However, the COX-2-dependent mechanisms are expected to have a relatively slow onset. For example, TNF-α enhances the sensitivity of sensory neurons to capsaicin via a COX-2-dependent pathway, which requires about 4 h to become effective [53]. In addition, acute TNF-α-mediated rapid onset hypersensitivity is COX independent [13]. We found that enhancement of ASIC currents by TNF-α was not blocked by indomethacin, a potent non-selective COX inhibitor. Thus, the COX-dependent mechanisms may not well explain the rapid effects of TNF-α on ASIC function. In addition, it has been reported that TNF-α trimers insert into the cell membrane to form a sodium-permeable ion channel under conditions of low pH (∼ pH = 5–6) [54, 55]. But we did not observed that TNF-α evoked any membrane currents in DRG neurons. Thus, we believed the rapid effects of TNF-α on ASIC function were mediated by its cognate receptors, but not by TNF-α ion channels.

Under inflammatory and neuropathic pain conditions, various mediators are released, such as ATP, prostanoids, protons, and TNF-α. They contribute to peripheral sensitization in terminals of nociceptor neurons [56]. Clinically, levels of TNF-α have been shown to elevate in several pain conditions [57]. Endogenous TNF-α and its receptors are upregulated in several models of pain [4, 5, 43]. Peripheral inflammation also results in p38 MAPK activation in nociceptive DRG neurons, which participates in inflammatory hyperalgesia [58]. Protons are released from damaged cells and the de-granulation of mast cells during inflammation, resulting in local acidification. These released protons are enough to activate ASICs [59]. Once both TNF-α and protons are locally released together at some sites, they could initiate and/or sensitize nociceptive process through activating their cognate receptors embedded in membrane of peripheral nerve terminals of nociceptive sensory neurons. Herein, we showed that the released TNF-α could rapidly enhanced ASIC-mediated and proton-activated currents in the same DRG neurons via a p38 MAPK pathway. In this work, we used cell bodies of DRG neurons as a simple and accessible model to examine the characteristics of the membrane of peripheral terminals. The sensitization of ASICs receptors by TNF-α may also occur in peripheral terminals. Behaviorally, we observed that TNF-α augmented acid-induced nociceptive responses in rats via activation of local p38 MAPK pathway. The TNF-α-induced rapid enhancement in ASIC-mediated electrophysiological activity could underlie exacerbation of nociceptive responses to acidification by TNF-α.

## Conclusion

In summary, our results indicated that acute application of TNF-α rapidly enhanced ASIC-mediated electrophysiological activity and acidosis-evoked pain, which revealed a novel peripheral mechanism underlying rapid sensitization to nociceptive stimuli by peripheral administration of TNF-α. ASICs represent downstream targets of TNF-α and therapy targeting ASICs is likely useful for treating inflammatory and neuropathic pain.

## Data Availability

Not applicable.

## Abbreviations

ANOVA: One-way analysis of variance
APs: Action potentials
ASICs: Acid-sensing ion channels
COX: Cyclooxygenase
DMEM: Dulbecco’s modified Eagle’s medium
DRG: Dorsal root ganglion
EC_50_: Half-maximal response
*I*_pH_: Proton-gated current
MAPK: Mitogen-activated protein kinase
PKA: Protein kinase A
PKC: Protein kinase C
TNF-α: Tumor necrosis factor-α
TNFR: Tumor necrosis factor receptor
TRPV1: Transient receptor potential vanilloid type 1
TTX: Tetrodotoxin
